# Increased sodium and fluctuations in minerals in acid limes expressing witches’ broom symptoms

**DOI:** 10.1186/s40064-016-2049-0

**Published:** 2016-04-06

**Authors:** Aisha G. Al-Ghaithi, Muhammad Asif Hanif, Walid M. Al-Busaidi, Abdullah M. Al-Sadi

**Affiliations:** Department of Crop Sciences, College of Agricultural and Marine Sciences, Sultan Qaboos University, Muscat, Oman; Department of Chemistry, University of Agriculture, Faisalabad, 38040 Pakistan

**Keywords:** Metals, Mexican lime, Oman, Pathogen, WBDL

## Abstract

Witches’ broom disease of lime (WBDL), caused by ‘*Candidatus* Phytoplasma aurantifolia’, is a very serious disease of acid limes. The disease destroyed more than one million lime trees in the Middle East. WBDL results in the production of small, clustered leaves in some branches of lime trees. Branches develop symptoms with time and become unproductive, until the whole tree collapses within 4–8 years of first symptom appearance. This study was conducted to investigate differences in minerals between symptomatic and asymptomatic leaves of infected lime trees. The study included one set of leaves from uninfected trees and two sets of infected leaves: symptomatic leaves and asymptomatic leaves obtained from randomly selected acid lime trees. Nested polymerase chain reaction detected phytoplasma in the symptomatic and asymptomatic leaves from the six infected trees, but not from the uninfected trees. Phylogenetic analysis showed that all phytoplasmas belong to the 16S rRNA group II-B. Mineral analysis revealed that the level of Na significantly increased by four times in the symptomatic leaves compared to the non-symptomatic leaves and to the uninfected leaves. In addition, symptom development resulted in a significant increase in the levels of P and K by 1.6 and 1.5 times, respectively, and a significant decrease in the levels of Ca and B by 1.2 and 1.8 times, respectively. There was no significant effect of WBDL on the levels of N, Cu, Zn, and Fe. The development of witches’ broom disease symptoms was found to be associated with changes in some minerals. The study discusses factors and consequences of changes in the mineral content of acid limes infected by phytoplasma.

## Background

Phytoplasmas are phloem limited, gram-positive bacteria in the *Mollicutes* class (Bové and Garnier 2002). They have been found associated with diseases in more than 700 plant species (Bertaccini et al. 2014; Hogenhout et al. 2008; Bertaccini 2007; Lee et al. 2000). Phytoplasma can cause different symptoms including phyllody, stunting, virescence, little leaf, yellowing, witches’ broom, decline, and other symptoms (Hogenhout et al. 2008). They are usually transmitted by sap-sucking insects of the order *Hemiptera*, especially by leafhoppers, planthoppers and psyllids (Sugio et al. 2011; Ammar and Hogenhout 2006; Hill and Sinclair 2000).

Witches’ broom disease, caused by *Candidatus* Phytoplasma aurantifolia, is a very serious disease of acid limes (*Citrus aurantifolia*). Symptoms of the disease are characterized by excessive shoot growth with very small, pale green leaves and short internodes. Witches’ broom symptoms progress very rabidly on lime branches, killing trees within 4–8 years after symptom appearance (Al-Sadi et al. 2012; Chung et al. 2006b). The disease resulted in the death of over one million lime trees in different countries (Chung et al. 2006a; Al-Sadi et al. 2012; Bertaccini and Duduk 2009; Al-Yahyai et al. 2014b).

Several factors can predispose plants to infection by microorganisms, including mineral imbalance (McNew 1953). Some diseases are severe on weakened and undernourished plants; others are more destructive when plants are growing vigorously. Plants need minerals for normal growth and development. Mineral elements play a significant role in the physiology and metabolisms of the plant and their characterization can reveal the genetics of the plants and their interaction with biotic and environmental factors (Baxter et al. 2013, 2014). Any deficiency or excess may affect plant growth, development and production. The severity of potato scab and club rot of cabbage increased with enhanced supply of calcium (Ca), potassium (K) or nitrogen (N) (McNew 1953).

Several studies have addressed the interaction between phytoplasma and their host plants. Phytoplasma have been shown to produce effectors to modulate their host plants and to overcome plant defense responses (Sugio et al. 2011; Sugio and Hogenhout 2012). Recent studies on the biochemical interactions showed that phytoplasma can affect essential oils of acid lime (Al-Yahyai et al. 2014a). A study by Rossi et al. (2010) showed Fe /Mn and K/Mg imbalance in phytoplasma infected pear and apricot, respectively. Zhao and Liu (2009) showed mineral nutrient differences between healthy and phytoplasma infected jujube, while De Oliveira et al. (2002, 2005) showed reduced concentrations of Mg in maize tissues infected by phytoplasma. However, there are few studies on the changes in minerals in acid limes developing symptoms of Phytoplasma infection.

This study was carried out to investigate changes in minerals in acid lime leaves developing symptoms of witches’ broom disease. Information on the mineral status of acid lime trees and the relationship between phytoplasma infection, symptom development and minerals concentration in plant tissues will help us understand some of the changes induced by phytoplasma in acid limes. This may help in the development of future management strategies for WBDL.

## Methods

### Sample collection

**Fig. 1:**
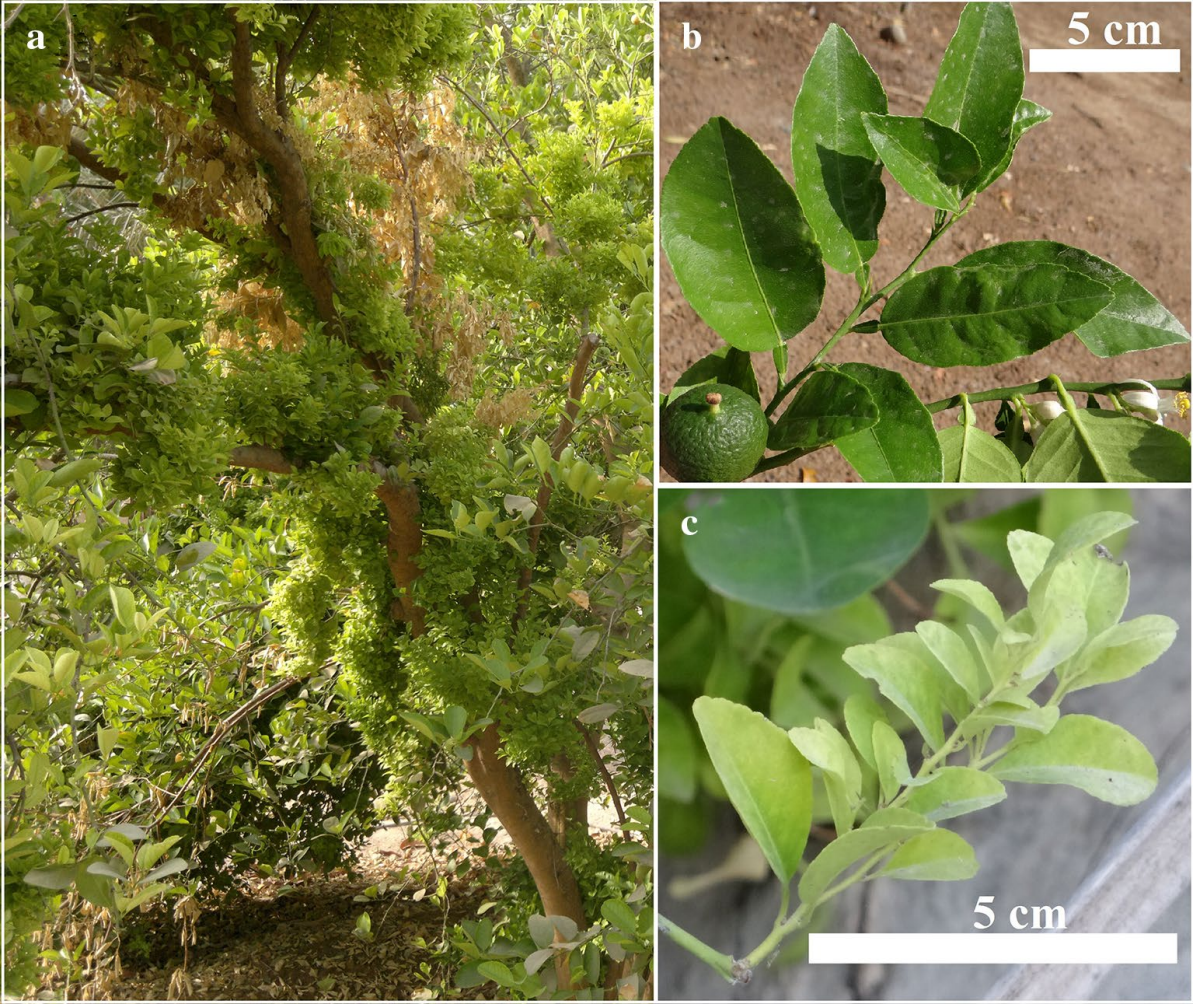
*Acid lime tree* infected by ‘*Candidatus* Phytoplasma aurantifolia’ and developing typical symptoms of witches’ broom disease (**a**). A close up photo of asymptomatic leaves (**b**) and symptomatic leaves with typical symptoms of WBDL (**c** smaller leaves, *light green* to *yellow* in color). The *bar scale* in (**b**, **c**) is 5 cm

Leaf samples of acid lime were collected from six lime trees developing symptoms of WBDL (Al-Sadi et al. 2012) (Table 1). Two types of samples were collected from each of the six trees: 1 kg of leaves without any disease symptoms and 1 kg of symptomatic leaves which are developing typical WBDL symptoms (i.e. small clustered leaves with dense and thin branches; Fig. 1). In addition, two samples were collected from uninfected lime trees for comparison purposes. The collected samples were transported to the Plant Pathology laboratory (SQU, Oman) in a cool box. The samples were dried at 70 °C, followed by grinding using a food processor (Moulinex, France) under sterile conditions. The obtained powdered materials were stored in airtight jars for further studies.

**Table 1:** Characteristics of acid lime trees and leaf samples included in the study

Tree code	Sample code	WBDL symptoms	Presence of phytoplasma^a^	Accession numbers^b^
YS1	YS1	No	−	−
YS2	YS2	No	−	−
D1	D1-A	Asymptomatic leaves	+	LN866569
	D1-S	Symptomatic leaves	+	LN866570
D2	D2-A	Asymptomatic leaves	+	LN866571
	D2-S	Symptomatic leaves	+	LN866572
B1	B1-A	Asymptomatic leaves	+	LN866565
	B1-S	Symptomatic leaves	+	LN866566
B2	B2-A	Asymptomatic leaves	+	LN866567
	B2-S	Symptomatic leaves	+	LN866568
SH	SH-A	Asymptomatic leaves	+	LN866579
	SH-S	Symptomatic leaves	+	LN866580
M1	M1-A	Asymptomatic leaves	+	LN866575
	M1-S	Symptomatic leaves	+	LN866576

^a^Based on PCR analysis, where (+) indicates presence of phytoplasma and (−) indicating the lack of phytoplasma

^b^16S rRNA sequences deposited at the European Nucleotide Archive

### Detection and identification of phytoplasma

Total DNA was extracted from 100 mg of the midrib from each sample following the cetyltrimethyl ammonium bromide (CTAB) method (Doyle and Doyle 1990). Primers P1 (5′-AAGAGTTTGATCCTGGCTCAGGATT-3′) (Deng and Hiruki 1991) and P7 (5′-CGTCCTTCATCGGCTCTT-3′) (Schneider et al. 1995) were used in amplification of phytoplasma 16S rRNA gene, spacer region between 16S and 23S rRNA genes and the start of 23S rRNA gene. The polymerase chain reaction (PCR) consisted of 35 cycles: denaturation at 94 °C for 30 s (2 min for the first cycle), annealing for 40 s at 60 °C and extension at 72 °C for 1.5 min (7.5 min for cycle 35). The PCR products were diluted 1:30 with sterile double-distilled water. Then samples were subjected to nested PCR using the general primer pair R16F2n/R2 (5′-GAAACGACTGCTAAGACTGG-3′/5′-TGACGGGTGTGTACAAACCCCG-3′) (Gundersen and Lee 1996). The PCR conditions consisted of denaturation at 94 °C for 2 min, followed by 35 cycles of denaturation at 94 °C (1 min), annealing at 60 °C (1 min) and primer extension at 72 °C for 1.5 min (7.5 min for cycle 35). PCR products from the direct and nested PCR were electrophoresed on 1.5 % agarose gel stained with ethidium bromide.

Nested PCR products were sequenced at Macrogen (Korea) using R16R2 and R16F2n primers. The forward and backward sequences of each sample were aligned and edited using Chromas Pro (version 1.41; Technelysium Pty Ltd, Brisbane, QLD, Australia). Then the resulting sequences were compared with reference sequences deposited at the National Center for Biotechnology Information using Mega 5 (Tamura et al. 2011). A phylogenetic tree was generated based on the matrix of pairwise distances using the Kimura 2 parameter evolutionary model (Mega 5), with 1000 replications and 50% bootstrap criteria (Hodgetts et al. 2008).

### Analysis of nitrogen in leaf samples

About 5 g of acid lime leaves from each sample were washed and freeze dried. Then, the dried leaves were ground to fine powder. Kjeldahl digestion method was used to determine the amount of N (Persson et al. 2008). About 3.5 ml of sulfuric acid was added to 0.1 g leaf sample. After that 3.5 g K_2_SO_4_ mixed with 0.4 g CuSo_4_·5H_2_O was added to the solution, followed by the addition of 3.5 g K_2_SO_4_ mixed with Se. Then, the mixtures were heated on hotplate at 350 °C for 4 h. Two blanks were prepared and treated as previously described. The digest was then analysed for total N using a Kjeltec Analyser.

### Analysis of sodium, potassium and calcium

One gram of leaf sample was wet digested using 20 ml HNO_3_ and 5 ml of H_2_O_2_ for 30 min at 150 °C. The final volume of the digested sample was made up to 50 ml in a volumetric flask using deionized distilled water (DDW). The samples were analyzed in triplicates (Ahmad et al. 2008; Jilani et al. 2012). The Na, K and Ca were analyzed using flame photometer (Sheerwood 450 flame photometer).

### Analysis of other macro and micro elements

Analysis of other macro and micro elements (B, P, Zn, Mg, Fe, Cu) in leaf samples was done following a modified protocol of Mihaylova et al. (2013). One gram of leaf sample was digested using 30 ml HNO_3_ and 10 ml of HCl for 30 min at 300 °C. The final volume of the digested sample from leaf samples was made up to 50 ml and 100 ml, respectively using DDW. The diluted mixtures were filtered by 0.45 µl filter membrane. Prepared samples were analyzed by inductively coupled plasma analysis **(**ICP-MS).

### Statistical analysis

The mean values of minerals leaf samples were analyzed for statistical differences using Tukey’s Studentized range test (SAS, v8, SAS Institute Inc., Cary, NC, USA).

## Results

### Identification of phytoplasma

Detection of phytoplasma in the 14 samples revealed presence of phytoplasma in all the symptomatic samples and asymptomatic samples from the six infected trees. No phytoplasma was detected in the disease-free uninfected trees. Direct and nested PCR resulted in the production of 1.8 k base pair and 1.25 kbp fragments, representing the 16S–23S rRNA gene and 16S rRNA gene of phytoplasma, respectively.

**Fig. 2:**
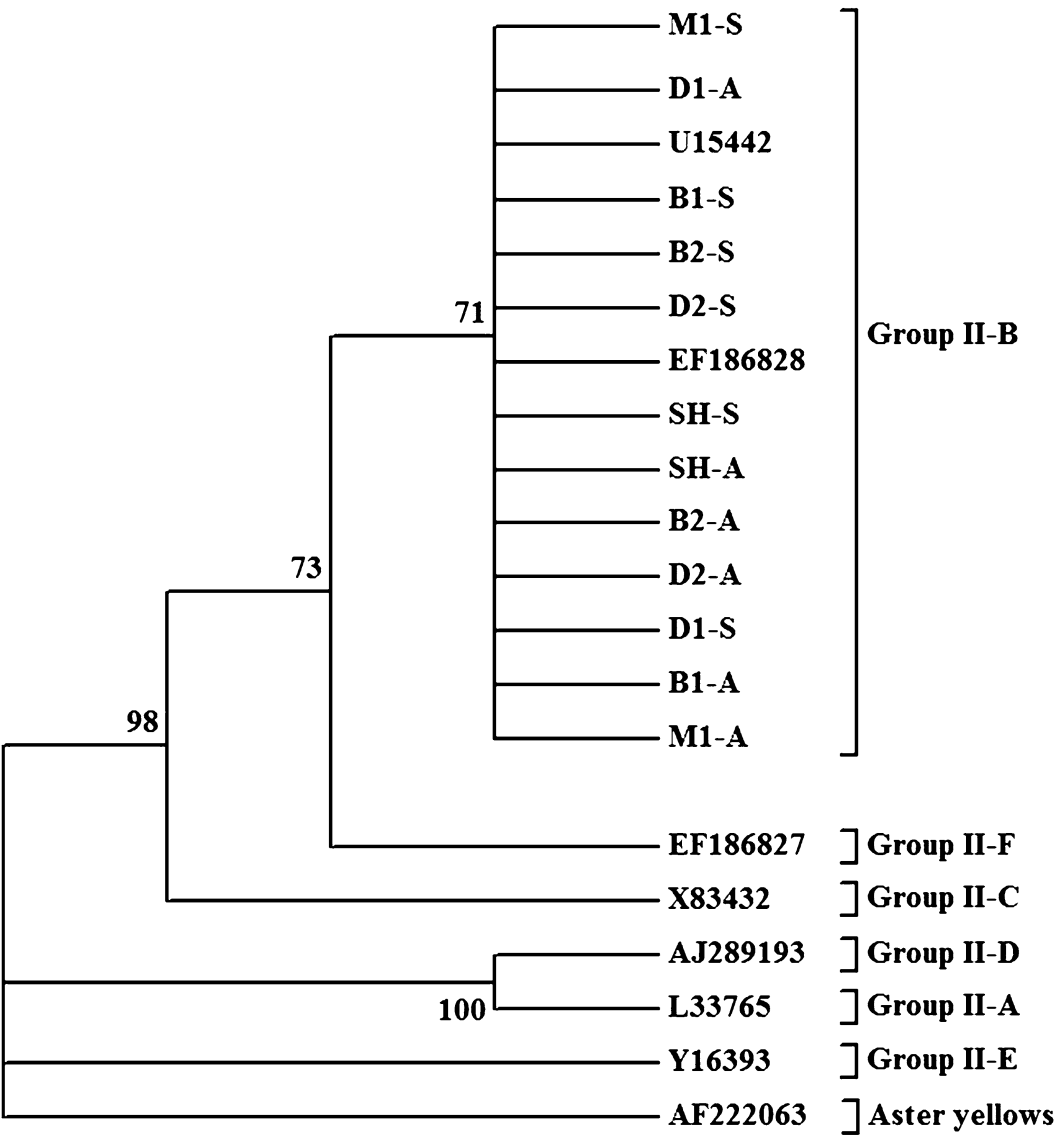
Phylogram representing the relationship of 12 phytoplasma isolates from Oman to 2 reference ‘*Candidatus* Phytoplasma aurantifolia’ accessions (U15442 and EF186828) from the 16S rRNA Group II-B and to other phytoplasma groups. The tree was rooted to *aster yellows* phytoplasma (AF222063). The 16S rRNA sequences of isolates from Oman were submitted to the European Nucelotide Archive under the accession numbers listed in Table 1. *Numbers within the tree* represent the bootstrap values (values above 70 % are indicated; 1000 replications). The tree is rooted to ‘*Ca*. P. asteris’ (16SrI) (*Aster yellows*) as outgroup

Analysis of 12 sequences representing the 16S rRNA gene of 12 samples showed that all sequences share 99.5–100 % sequence similarity to each other and also to a previously deposited sequence of ‘*Ca*. Phytoplasma aurantifolia’ from lime (EF186828) (Fig. 2). The 16S rRNA sequences of the 12 samples were deposited at European Nucleotide Archive under the accession numbers listed in Table 1.

### Mineral analysis in leaves

**Fig. 3:**
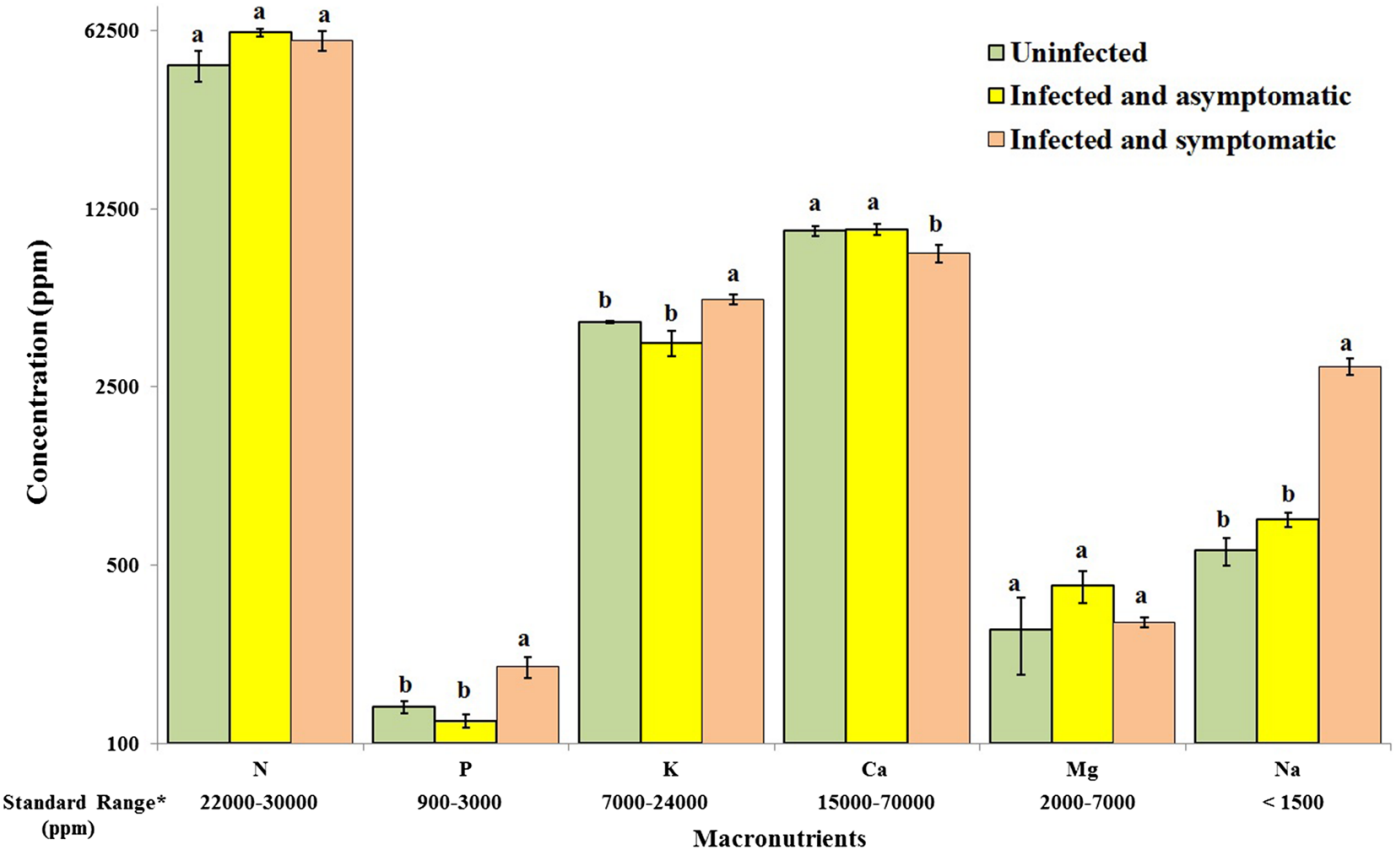
Macro-element composition of uninfected, infected asymptomatic and infected symptomatic acid lime leaves. The *data below* *the figure* represent the standard range of elements according to (Yen 1972) and (Obreza et al. 2004). Values with the *same letter* in the *same mineral* category are not significantly different from each other at P < 0.05 (Tukey’s Studentized range test, SAS). The Y-axis is logarithmic scale. The *number* of biological and technical replicates was 2 and 5 for the control, 6 and 18 for the infected asymptomatic leaves and 6 and 18 for the infected symptomatic leaves, respectively

Mineral analysis showed that there is no significant difference in the concentration of minerals between the uninfected leaf samples and the infected leaf samples which are not developing typical symptoms of WBDL (P > 0.05). However, the concentration of Na was found to be significantly higher by four times in the infected leaves developing typical symptoms of WBDL (3008 ppm) compared to the infected but asymptomatic leaves (757 ppm) collected from the same trees (Fig. 3; P < 0.05). The concentration of Na in the uninfected samples was found to be 570 ppm, falling within the range of the asymptomatic leaves which are infected by phytoplasma. The concentration of P and K also followed the same trend as Na, but the concentrations were significantly higher by 1.6 times for P and 1.5 times for K in the symptomatic leaves compared to the asymptomatic leaves and the uninfected leaves (Fig. 3; P < 0.05).

**Fig. 4:**
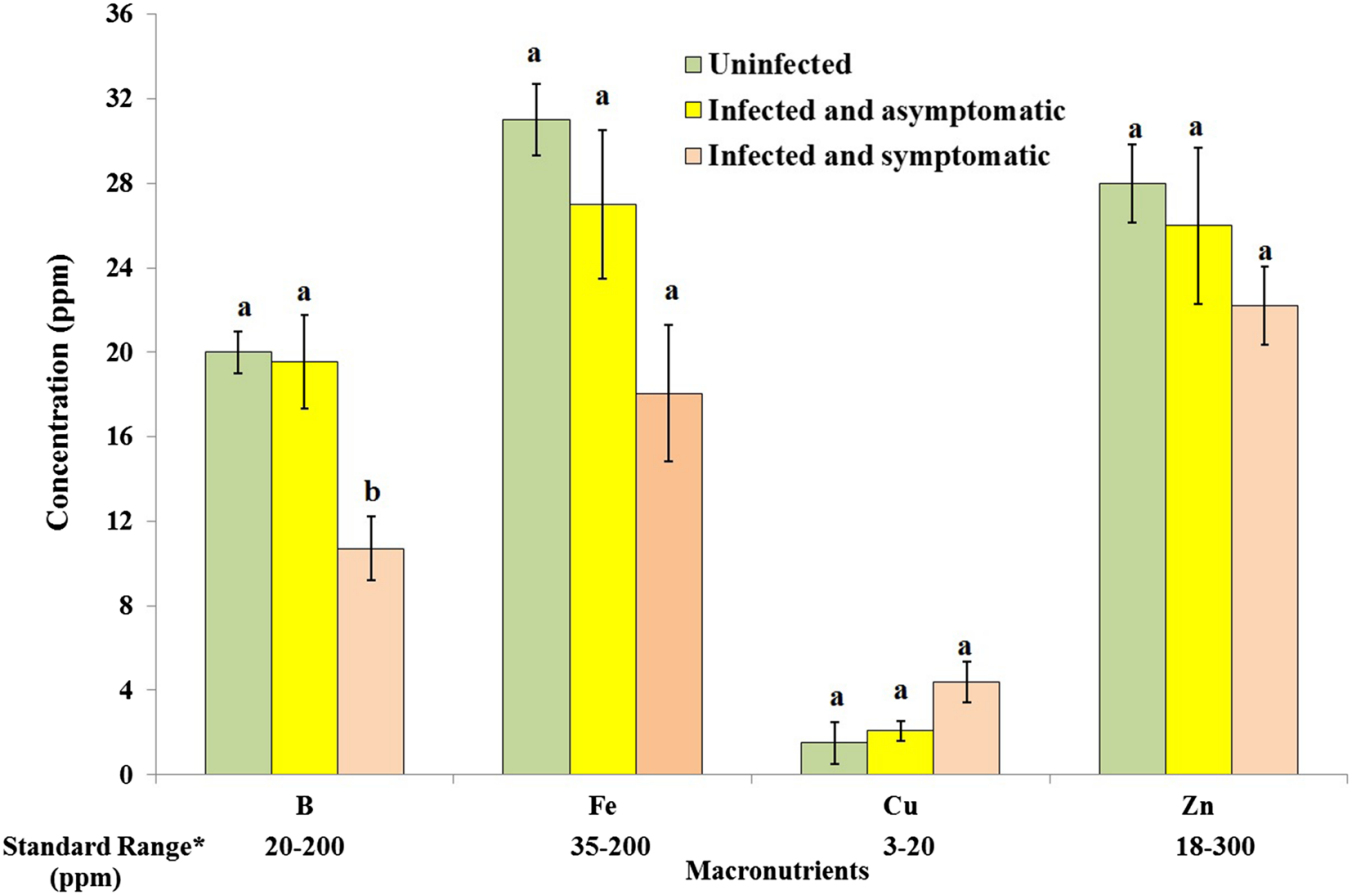
Micro-element composition of uninfected, infected asymptomatic and infected symptomatic acid lime leaves. The *data below the figure* represent the standard range of elements according to (Yen 1972) and (Obreza et al. 2004). Values with the *same letter* in the *same mineral* category are not significantly different from each other at P < 0.05 (Tukey’s Studentized range test, SAS). The *number* of biological and technical replicates was 2 and 5 for the control, 6 and 18 for the infected asymptomatic leaves and 6 and 18 for the infected symptomatic leaves, respectively

On the other hand, the concentrations of Ca and B followed an opposite trend to that of Na, P and K. The concentrations of Ca and B were significantly lower by 1.2 and 1.8 times, respectively, in the symptomatic leaves compared to the asymptomatic leaves and the uninfected leaves (Figs. 3, 4). There were no significant differences between the symptomatic and asymptomatic leaves in the levels of N, Mg, Fe, Cu, and Zn (Figs. 3, 4).

Analysis of minerals in lime leaves showed that the uninfected and infected acid lime leaves have higher levels of N compared to standards described by Yen (1972) and Obreza et al. (2004) (Fig. 3). The level of Na was within the standard range for the asymptomatic leaves as well as the uninfected leaves. However, the level of Na was excess in infected leaves developing symptoms of WBDL. The levels of B and Zn in the uninfected and asymptomatic leaves were within the standards. The levels of P, K, Ca, Mg, Fe, and Cu were below the satisfactory range in the uninfected, symptomatic and asymptomatic leaves (Figs. 3, 4).

## Discussion

Mineral analysis showed that the level of Na in the asymptomatic leaves as well as the uninfected leaves was within the acceptable levels. However, symptomatic leaves of acid limes accumulated approximately 4 times more Na, 1.6 times more P and 1.5 times more K compared to the asymptomatic leaves of the same trees. The accumulated levels of Na were above the recommended limit of 1500 ppm (Obreza et al. 2004), reaching 3008 ppm. Excess amounts of Na may result in defoliation, leaf burn and dieback (Storey and Walker 1999), which have already been observed and reported in phytoplasma infected acid limes in Oman (Chung et al. 2006a). Thus the accumulation of Na in the symptomatic leaves appears to play a role in defoliation and dieback symptoms due to WBDL. This appears to be the first record of accumulation of Na in the symptomatic branches of acid limes infected by ‘*Ca*. Phytoplasma aurantifolia’. Future studies are required to investigate the mechanisms driving the accumulation of Na in the symptomatic branches of WBDL infected limes. The levels of P and K also increased in the symptomatic leaves, but they were still below the recommended levels in leaves (Yen 1972; Obreza et al. 2004). Mohan and Rao (1986) reported that P accumulated in high amount in banana with bunchy top disease compared to healthy plants. Potassium is essential for basic physiological functions such as the formation of sugars and starch, synthesis of proteins, and cell division and growth. It is also important in fruit formation and enhances fruit flavor, size and color. Potassium also helps to normalize the CO_2_ supply to citrus trees by controlling the opening and closing of stomata. Therefore, the rate of photosynthesis drops sharply when plants are K deficient. Potassium increases plant resistance to disease, size and quality of fruit and winter hardiness (Obreza 2003).

Findings from this study showed that symptom development due to WBDL was associated with significant reductions in the concentrations of Ca and B by 1.2 and 1.8 times, respectively. Calcium is an essential part of cell walls and membranes, and must be present for formation of new cells. The ratio of Ca and K was reported by McNew (1953) as important factor in the mobilization of cationic nutrients. In addition, it was observed that potato scab infection increased as supplies of Ca were increased in the presence of inadequate supply of K (McNew 1953). Boron deficiency was reported to result in dieback symptoms (Chapman 1989), which has been observed on lime trees infected by phytoplasma in Oman (Al-Sadi et al. 2012).

No significant differences were found between symptomatic and asymptomatic leaves in the levels of N, Mg, Cu, Fe and Zn. This suggests the lack of influence of symptom development due to WBDL on these minerals. However, the levels of N were generally high compared to standard levels in citrus leaves. Excessive N levels cause nitrogen toxicity and leaves eventually turn yellow or brown and fall off (Purcino et al. 2007).

Mineral analysis in acid lime leaves, especially the uninfected leaves, revealed that the levels of P, K, Ca, Mg, Fe and Cu are different from the standards reported by Yen (1972) and Obreza et al. (2004). Han et al. (2011) reported that the concentration of minerals in plant leaves can vary according to climate, soil type and plant functional type. In addition, the majority of growers in Oman use animal manures for fertilizing the acid lime trees, without paying attention to the exact amounts of minerals required by acid limes. It is therefore likely that a combination of these factors affected the levels of minerals in acid lime leaves in Oman.

## Conclusion

This study is the first to characterize the relationship between symptom development and mineral concentration in acid limes infected by ‘*Ca*. Phytoplasma aurantifolia’. Development of symptoms due to WBDL was found to be associated with mineral imbalance, especially on the levels of Na in the symptomatic leaves. Future studies should address the mechanisms driving the mineral imbalance in acid limes developing symptoms of WBDL. Further research is required to elucidate the roles of significantly affected minerals in the susceptibility/resistance of acid limes to “*Ca*. Phytoplasma aurantifolia”, and to determine how strategies might be developed to overcome such changes using chemicals or other treatment of lime trees.
